# The Continuity Trap in Data Science Health Research

**DOI:** 10.2196/98699

**Published:** 2026-08-19

**Authors:** Clement Adebamowo, Sally Nneoma Adebamowo, Adeola Akintola, Peter Ikhane, Simisola Akintola, Temidayo Ogundiran, Ayodele Jegede, Olusegun Adeyemo, Shawneequa Callier, Muhammad Imam-Tamim, Ibrahim Uthman, BridgELSI Project as part of the DS-I Africa Consortium

**Affiliations:** 1 Epidemiology and Public Health School of Medicine University of Maryland, Baltimore Baltimore, MD United States; 2 University of Maryland Marlene and Stewart Greenebaum Comprehensive Cancer Center Baltimore, MD United States; 3 Department of Research Center for Bioethics and Research Ibadan, Oyo Nigeria; 4 Department of Bioethics and Medical Humanities Faculty of Multidisciplinary Studies University of Ibadan Ibadan, Oyo State Nigeria; 5 Faculty of Law University of Ibadan Oyo, Oyo State Nigeria; 6 Department of Surgery College of Medicine University of Ibadan Oyo, Oyo State Nigeria; 7 Department of Sociology Faculty of Social Sciences University of Ibadan Ibadan, Oyo State Nigeria; 8 Department of Clinical Research and Leadership School of Medicine and Health Sciences George Washington University Washington DC, DC United States; 9 Department of Islamic Law Faculty of Law University of Ilorin Ilorin, Kwara State Nigeria; 10 Department of Arabic and Islamic Studies Faculty of Islamic Studies University of Ibadan Ibadan Nigeria

**Keywords:** concept drift, data life cycle, dataset shift, ethical governance continuity, machine learning governance, management, model accountability

## Abstract

Secondary use is now the ordinary condition of data science health research rather than an exception to it. Electronic health records collected for clinical care become prediction tools and inputs for generative AI; imaging archives become foundation-model corpora; genomic datasets become resources for polygenic risk scores; and legacy biospecimens become renewable, indefinitely distributable cell lines. Governance has responded by emphasizing verifiable instruments such as provenance logs, repository approvals, broad-consent forms, data-use agreements, model cards, records of processing, and locality-preserving architectures. These instruments are necessary, and they answer real questions about lineage, privacy, institutional responsibility, and accountability, but they are not sufficient to establish that a present use remains ethically justified. We define ethical continuity as the persistence of normatively relevant relationships between the original conditions of data generation or material collection and subsequent downstream uses, such that current uses remain justifiable in light of the expectations, permissions, meanings, and relational obligations present at entrustment. We then define the Continuity Trap as a review-stage governance error in which a salient signal of continuity in one domain is treated as sufficient evidence of ethical continuity overall, causing inquiry into the remaining domains to close prematurely. The trap is not ordinary noncompliance, ethics creep, or a demand for universal rereview; it is a cross-domain inference error that can arise even in careful, good-faith review. We distinguish it from proxy closure, of which it is a continuity-specific subtype, and from Goodhart’s and Campbell’s laws, which describe how measures degrade once they become targets. We operationalize ethical continuity across 4 domains: provenance, semantics, authorization, and relational standing, developed in our Representational Veracity framework, and we show that these domains can diverge as data are linked, transformed, modeled, and redeployed. We identify the institutional mechanisms—provenance privilege, descriptor sedimentation, authorization fossilization, and community effacement—that cause auditable signals to be overread, and we examine how the US Health Insurance Portability and Accountability Act (HIPAA) of 1996, the General Data Protection Regulation, the European Health Data Space, US Food and Drug Administration guidance, the US National Institute of Standards and Technology (NIST) AI Risk Management Framework, and federated-learning governance can reduce risk while still inducing continuity traps. We apply the framework to consent and nonconsent settings, including public health, immunization, syndromic, and wastewater surveillance, polygenic risk scores, induced pluripotent stem cells, federated learning, and health-related large language models. The policy implication is trigger-based continuity review: rather than rereviewing every reuse, investigators and reviewers should identify the weakest continuity domain at the present data stage and impose a domain-matched safeguard, recorded in a short continuity statement. This reframing is intended for the committees, repositories, funders, and governance bodies that decide whether reuse may proceed, and it matters most in cross-border and low-resource settings. Provenance should begin ethical review; it should not end it.

## Introduction

Data science health research increasingly depends on data and materials generated for other purposes. Clinical records become prediction models; images become foundation-model corpora; genomic datasets become inputs for polygenic risk scores; and stored biospecimens become cell lines or other derivatives. Secondary use is therefore not an exception to the field but one of its ordinary conditions, driven by the falling cost of storage, the reach of linkage and deidentification methods, and the appetite of contemporary machine-learning systems for large and heterogeneous training corpora. Recent discussions of consent for secondary use in AI models, clinical machine-learning ethics, health-equity harms, privacy in the age of medical big data, the ethics of sharing clinical imaging data, and dataset and model documentation all converge on the same point: legal access and technical documentation are indispensable, but they do not exhaust the ethical inquiry [1-11]. Governance frameworks grew up around these auditable artifacts because they can be produced, filed, and inspected, which is also why they come to stand in for the harder judgments they cannot make.

The ethical governance difficulty that arises from this is that the most visible forms of continuity are often the easiest to overread. An institutional review board, data-access committee, repository, material-transfer office, or AI governance body can verify a consent form, access approval, chain-of-custody record, data-use agreement, source-data statement, model card, or locality-preserving architecture. These instruments answer important questions about lineage, privacy, institutional responsibility, and accountability, and they are frequently the only artifacts a reviewer can inspect within the time and authority available to a single review episode. They do not, by themselves, show that the present use remains ethically continuous with the conditions under which persons, communities, clinicians, public institutions, and source organizations entrusted data or material to health research. Careful reviewers acknowledge as much, yet the structure of review tends to reward the signals that can be filed and audited over those that must be interpreted [5-7,12,13]. The asymmetry is structural rather than a matter of individual diligence: filed artifacts survive audit, while interpretive judgments about meaning and standing rarely leave a trace that a later inquiry can reward.

*Ethical continuity* refers to the persistence of normatively relevant relationships between the original conditions of data generation or material collection and subsequent downstream uses, such that current uses remain justifiable in light of the expectations, permissions, meanings, and relational obligations under which the original collection occurred. This definition is deliberately broader than consent. It includes consent where consent is ethically and legally relevant, but it also includes public justification, statutory authority, proportionality, semantic validity, community expectations, and relations of trust. Framing the question as one of continuity rather than compliance keeps attention on the substantive relationship that governance is meant to protect, and it aligns with recent arguments that ethics frameworks for big data and for AI must attend to more than the presence of principles or paperwork [14-16]. *Continuity* is therefore a property of a relationship over time, not of a document, and it can lapse even where every document remains valid.

The aim of this viewpoint is to define and operationalize the Continuity Trap in data science health research. The intended audience is those who decide whether secondary use may proceed. They include research ethics committees, data-access committees, repositories, health-system AI governance bodies, funders, journals, regulators, investigators, and developers. Our central claim is that ethical continuity is domain-specific. It must be assessed across provenance, semantics, authorization, and relational standing domains. The operational goal is not to require full rereview for every request for secondary data use. It is to prevent one visible continuity signal from closing inquiry into the other domains too early. We proceed by situating the trap among adjacent governance critiques, defining it precisely, specifying its 4 domains and the institutional mechanisms that produce it, testing it against regulatory frameworks and a set of consent and nonconsent vignettes, and setting out a proportionate policy response (Textbox 1). This formulation builds on the Representational Veracity framework, institutional sociology, audit studies, contextual integrity, and the AI and data-ethics literature on life cycle harms, documentation, fairness, and governance [8,13,14,17-19]. The structure of the argument is cumulative: each later section presupposes the domain distinctions introduced earlier, and the vignettes are meant to test the framework rather than merely illustrate it.

**Key terms and concepts.**

**Ethical continuity**

- Persistence of normatively relevant relationships between original collection conditions and later uses, such that current uses remain justifiable in light of expectations, permissions, meanings, and relational obligations present at entrustment.

**Continuity Trap**

- A review-stage governance error in which a salient signal of continuity in one domain is treated as sufficient evidence of ethical continuity overall, causing inquiry into other continuity domains to close prematurely.

**Continuity signal**

- A visible artifact or feature, such as provenance, broad consent, repository approval, locality, or model documentation, that suggests continuity in one domain but may not establish ethical continuity overall.

**Cross-domain closure**

- An inference error in which continuity in one domain closes inquiry into other domains, including semantics, authorization, relational standing, or provenance itself.

**Provenance continuity**

- Continuity of lineage, handling, access history, transformation, and traceability between earlier and present data-stages.

**Semantic continuity**

- Continuity between labels, descriptors, ontologies, variables, and the constructs or populations they are taken to represent.

**Authorization continuity**

- Continuity between the current use and the permissions, expectations, legal authorities, and justificatory scope under which data or material were originally entrusted or collected.

**Relational standing**

- The continuing capacity of affected persons, communities, or source institutions to contest, shape, govern, or share in value when downstream uses create group-level claims, risks, or benefits.

**Data-stage**

- The specific state of a dataset, biospecimen, model, derivative, embedding, cell line, or deployment at the point of review.

**Domain-matched safeguard**

- A safeguard chosen because it addresses the weakest continuity domain, rather than adding generic paperwork.

## Existing Governance Problems

The Continuity Trap is related to established critiques of governance, but it does not collapse into any of them (Table 1). Proxy closure is the broader genus: a partial and administratively convenient proxy is treated as if it exhausts a more complex moral or institutional object. In health data science, a provenance log can stand in for ethical legitimacy, a consent document can stand in for present authorization, and a model card can stand in for accountability. The proxy may be accurate as far as it goes, but the error lies in treating it as complete. What distinguishes the Continuity Trap from proxy closure in general is its object and its timing. The proxied object is specifically the continuity of an entrustment relationship over time, and the closure occurs at an identifiable review episode where a decision to proceed is made [5,6,17,20]. A repository approval, for instance, records that access was granted under stated terms, but it says nothing about whether those terms still fit a use that has since changed in purpose or population.

**Table 1:** The Continuity Trap compared with adjacent governance concepts.

Concept	What is overread or optimized?	Unit of failure	Why it matters for secondary use
Proxy closure	A partial proxy is treated as if it exhausts a complex value.	Any governance or evaluative setting.	Broader genus: legibility substitutes for fidelity.
Continuity Trap	A continuity signal in one domain is treated as sufficient evidence of ethical continuity overall.	Secondary-use review episode.	Continuity-specific subtype: provenance, broad consent, locality, or documentation closes inquiry into semantics, authorization, and relational standing.
Goodhart/Campbell dynamics	A measure becomes a target, and optimization degrades its relation to the underlying goal.	Incentive or compliance regime.	Dynamic intensifier once continuity signals become targets.
Ethics creep	Ethics review expands to more activities and contexts.	Institutional jurisdiction.	Important background risk, but the trap concerns premature closure within review.
Organizational decoupling	Formal compliance structures persist while substantive practice is weakly connected to them.	Organizational practice.	Explains how continuity review can appear complete while the relevant relation remains underexamined.

Goodhart’s and Campbell’s laws describe a related dynamic in which, once a measure becomes a target, actors optimize toward the measure and weaken its relation to the value it was supposed to track. A health system rewarded for complete documentation may produce excellent model cards while leaving unresolved whether descriptors are meaningful, whether public-health data have been repurposed beyond their justification, or whether source communities have recourse. These dynamics intensify the Continuity Trap once provenance completeness, locality preservation, or documentation becomes the compliance target, because the very thoroughness of the auditable record can make the unexamined domains harder to see. The 2 ideas are nonetheless distinct. Goodhart and Campbell describe the gradual corruption of a measure under incentive pressure, whereas the Continuity Trap can occur in a single, good-faith review in which no measure has yet been gamed [21,22]. In continuity terms, the danger is that a target once adopted for good reasons quietly redefines an ethical question as a documentary one.

Ethics creep and organizational decoupling explain the institutional background. Ethics creep concerns the expansion of review into more activities and contexts; organizational decoupling describes the separation between formal compliance structures and actual practice, in which organizations maintain the appearance of conformity while substantive practice drifts. The Continuity Trap is a narrower concept than either of these. It names an inference failure inside a review episode, where the review closes because one continuity signal is asked to do governance work that properly belongs to other domains. It is compatible with a review system that is neither overextended nor decoupled, which is why naming it adds something the existing critiques do not already supply [18,19,23,24]. Keeping the concept narrow is deliberate, because a diagnosis that applies everywhere offers reviewers no guidance about where to look first.

## Defining the Continuity Trap

The Continuity Trap is a continuity-specific form of proxy closure, and it has 3 features. First, the proxied object is ethical continuity in secondary data use, and not quality, fairness, privacy, or compliance in the abstract. Second, the unit of failure is the review episode: an access decision, repository approval, derivation decision, transfer authorization, model-release decision, or deployment clearance. Third, the error is cross-domain closure. Continuity in one domain, most commonly the provenance domain, but sometimes consent, repository approval, locality, or model documentation, is treated as sufficient reason to stop inquiry into the other domains. Understood this way, the trap is a systems-level failure of inference rather than a failure of any single instrument, which is why remedies aimed only at improving the instrument tend to leave it intact [20,25]. Because the failure is one of inference rather than of any single record, adding another record rarely dislodges it; what changes the outcome is a reviewer asking which domain the visible signal has been allowed to speak for.

This definition avoids 2 unhelpful responses. The first is to assume that any secondary use requires full rereview. That would be impractical, would slow legitimate research, and would disadvantage institutions with fewer administrative resources. The second is to assume that legal access or adequate documentation settles the ethical question. That approach is administratively attractive but ethically thin. The Continuity Trap occupies the space between these positions. Reviewers should identify when the present data-stage has changed enough that continuity must be reexamined, and should specify which domain is weak, rather than defaulting either to blanket rereview or to documentary sufficiency [26,27]. The middle position asks more of reviewers than a checklist and less than a full rereview, which is what makes it usable under real institutional constraints.

**Figure 1:**
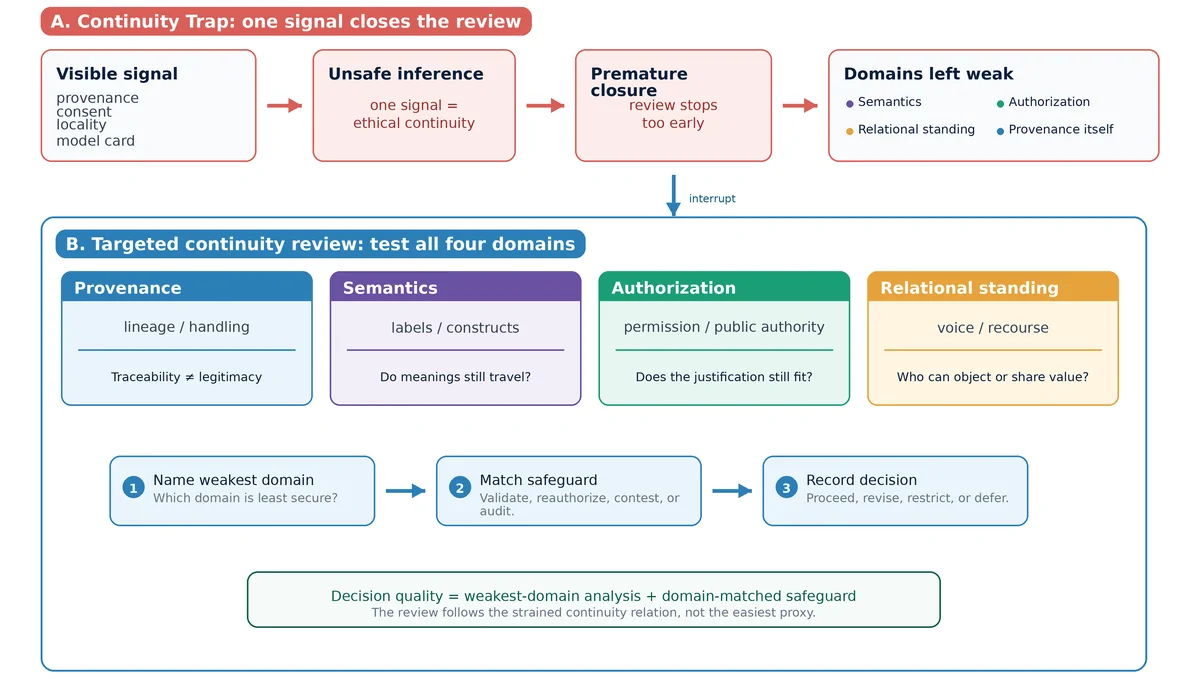
Inferential structure of the Continuity Trap.

Figure 1 summarizes the inferential structure. A visible continuity signal may be accurate in its own domain, but the inference from that signal to ethical continuity overall is unsafe unless the other domains have also been assessed. The corrective action is a targeted one. Identify the weakest domain and require a safeguard that addresses that domain.

## The 4 Domains of Ethical Continuity

Ethical continuity is not monolithic. It is relation-specific across 4 domains: provenance, semantics, authorization, and relational standing, developed in the Representational Veracity framework [14]. The data-stage may be a linked dataset, a deidentified file, a trained model, an aggregate embedding space, a derived cell line, a commercial derivative, or a deployment. The governance question is whether the relationships that made earlier collection or use justifiable remain strong enough at the later data-stage to support the decision being made now. Separating the domains is what allows a reviewer to say precisely where continuity has weakened, instead of reaching a single undifferentiated judgment that the use is or is not acceptable [14]. A dataset that was semantically coherent when collected for one clinical purpose can become semantically unstable once it is linked, harmonized across sites, or repurposed as a model input, even though its provenance record remains unbroken.

The domains can diverge, and it is their divergence that the trap exploits. A model may be traceable yet semantically unstable. A dataset may be legally accessible yet relationally extractive. A deidentified file may satisfy a privacy standard yet remain vulnerable to reidentification through linkage or generative inference, so that provenance and privacy assurances mislead about residual risk. A public-health registry may be authorized for surveillance but unauthorized for immigration enforcement. A federated architecture may preserve locality while transforming clinical records into training inputs for a commercial model. A model card is relevant evidence for documentation and accountability, but it is not relational standing; a consent form is relevant to authorization, but it is not authorization continuity itself [13,28,29]. The point is not that any one signal is untrustworthy, but that a signal true in its own domain licenses no inference about the others without independent examination (Table 2).

**Table 2:** Domains of ethical continuity and proxies that commonly mislead review.

Domain	What it tracks	Visible proxy that can mislead	Load-bearing review question
Provenance	Lineage, handling, access history, and transformations.	Audit trail, repository approval, data-use agreement, ingestion log.	What does traceability establish, and what does it not establish, about meaning, authorization, and accountability?
Semantics	Descriptors, ontologies, labels, variables, and inferred constructs.	Stable variable names, harmonized labels, ancestry labels, model-feature names.	Do current categories still map onto the same constructs, persons, populations, or groups?
Authorization	Permissions, expectations, statutory authority, public justification, proportionality, and justificatory scope.	Broad consent, legacy IRB^a^ approval, public-health authority, license terms, emergency exemption.	Is the present use still within the justified scope of entrustment or public authority?
Relational standing	Voice, recourse, contestability, governance participation, and claims to benefit when group-level claims or harms arise.	Naming a source group, token consultation, diversity language, community acknowledgment.	Who can object, shape conditions, participate in oversight, or share in value?

^a^IRB: institutional review board.

## Operational Conditions for Relational Standing

Relational standing should not be invoked whenever individual consent is thin. It becomes governance-relevant when 4 conditions are met: group-level claims are foreseeable; an affected collectivity is reasonably identifiable; likely harms or benefits are nontrivial; and a plausible route to representation exists or can be constructed. The fourth condition requires care. Communities with weak organizational infrastructure should not lose standing because they lack formal representatives, since that would penalize precisely the groups most exposed to extractive reuse. When the first 3 conditions are present but no representational route exists, that absence should itself be documented as a governance finding rather than used to bypass review [26,27,30]. Constructing a route to representation, where none yet exists, is itself a legitimate governance task rather than a precondition that affected groups must satisfy on their own.

This approach is especially important in cross-border and low-resource settings, where data and materials often move from communities with little bargaining power to institutions with substantial analytic and commercial capacity. Communitarian moral philosophy gives the relational domain a normative foundation by emphasizing identification, solidarity, reciprocal recognition, and shared benefit. On that view, extracting value from community-generated data without practical voice, benefit, or recourse is not only an autonomy problem. It also weakens the relationships through which data, identity, and social meaning were constituted, and it corrodes the trust on which future participation depends. Accounts of solidarity in biomedicine make the same point from a different direction, treating willingness to share in one another’s costs as a resource that governance can either sustain or deplete [31-35]. Where benefit and voice are absent, what appears to be permissible reuse can amount to a transfer of value away from the very people whose lives the data describe.

## Institutional Mechanisms That Produce the Continuity Trap

The Continuity Trap is produced by recurrent institutional mechanisms that give disproportionate weight to what is most auditable. Provenance privilege arises because lineage is legible, serializable, and defensible in post hoc accountability inquiries. Descriptor sedimentation occurs when old labels become embedded in forms, repositories, metadata schemas, and analytic pipelines, so that categories persist long after the constructs they name have shifted. Authorization fossilization occurs when formal permissions outlive the ethical coherence of the uses they are asked to justify. Community effacement occurs when groups remain visible as sources of diversity, legitimacy, biological material, or epidemiological signal but disappear as participants in downstream governance. Each mechanism is a rational response to institutional incentives, which is why exhortation alone does not remove them [17,18,26,33,34]. None of these mechanisms requires bad faith; each is the predictable result of asking reviewers to account for their decisions using the artifacts an institution is equipped to store and defend.

These mechanisms reinforce one another. What can be documented receives more institutional weight than what must be interpreted, negotiated, validated locally, or sustained over time, and the accumulation of auditable records can itself become the evidence that further inquiry is unnecessary. The Continuity Trap is the point at which that preference hardens from a habit into a decision rule, so that the presence of a strong record in one domain is taken, without further argument, to license the decision to proceed [17,18]. At that point the record has stopped serving inquiry and has begun to substitute for it.

## Regulatory and Governance Context

Regulatory and governance frameworks can reduce risk and still induce continuity traps. US Health Insurance Portability and Accountability Act (HIPAA), the General Data Protection Regulation, the European Health Data Space, US Food and Drug Administration guidance for AI-enabled device software, the US National Institute of Standards and Technology (NIST) AI Risk Management Framework, the National Academy of Medicine Artificial Intelligence Code of Conduct, World Health Organization guidance, institutional health-AI governance, and federated-learning arrangements all emphasize accountability, security, traceability, life cycle management, or responsible use in different ways [36-46]. These are real strengths, and nothing in the following argument is a case for weakening them. The risk is that institutions optimize toward what regulators and auditors make most explicit, and treat satisfaction of an explicit requirement as though it answered questions the requirement was never designed to reach. The same explicitness that makes a regime auditable can also narrow the questions a reviewer feels authorized to ask, so that what falls outside the checklist falls outside the review.

This regulatory critique is deliberately modest. HIPAA is not a theory of semantic validity; Food and Drug Administration device guidance is not a community-governance code; the NIST framework is not a substitute for human-participant review; and World Health Organization surveillance guidance is not a license for unrelated enforcement use. The problem arises when compliance with one regime is treated as if it settled continuity relations that the regime was not designed to evaluate. A complete processing record, a valid waiver, a secure-aggregation protocol, or a model-governance template may be accurate and still leave the central ethical question unresolved. Work on regulating adaptive and continually learning systems, and on taking a system rather than a product view of medical-device software, makes a parallel point: governance has to track the object as it changes, not only certify it at a moment of approval [25,47,48]. Governance that certifies an object once, and treats the certificate as durable, is poorly matched to systems whose purpose, population, and behavior continue to change after approval (Table 3).

**Table 3:** Examining governance regimes through a continuity lens.

Regime or framework	Primary governance strength	Continuity risk	Supplement needed
HIPAA^a^/public health authority	Permitted disclosures, authorization, waiver, deidentification, data-use agreements, and public-health disclosure rules.	Legal permission may be mistaken for semantic or relational continuity.	Review purpose shift, descriptor validity, and downstream accountability beyond disclosure legality.
GDPR^b^/EHDS^c^	Lawful basis, accountability, records of processing, rights, access, and regulated secondary use of electronic health data.	Processing records may document activity without resolving group-level meaning or standing.	Embed continuity questions into records, impact assessments, access decisions, and transfers.
FDA^d^ AI-enabled device guidance	Life cycle management, planned modifications, validation, and safety/effectiveness assessment.	Technical monitoring may not identify authorization drift or descriptor sedimentation.	Continuity review at modification and deployment-change points.
NIST^e^, NAM^f^, and WHO^g^ AI guidance	Risk management, responsible and equitable health AI, impacted-person engagement, and LMM-specific governance.	High-level principles may not expose review-stage cross-domain closure.	Domain-specific continuity prompts within AI governance workflows.
Federated-learning governance	Locality preservation, privacy-preserving computation, aggregation controls, and distributed infrastructure.	“Data stayed local” may be mistaken for authorization, semantic, and relational adequacy.	Site-level semantic validation, purpose review, benefit-sharing, and contestability.

^a^HIPAA: US Health Insurance Portability and Accountability Act of 1996.

^b^GDPR: General Data Protection Regulation.

^c^EHDS: European Health Data Space.

^d^FDA: US Food and Drug Administration.

^e^NIST: US National Institute of Standards and Technology.

^f^NAM: US National Academy of Medicine.

^g^WHO: World Health Organization.

## Authorization Continuity Beyond Consent-Based Frameworks

Authorization continuity is often read too narrowly as consent continuity. Public health surveillance shows why that reading is incomplete. Surveillance, disease notification, immunization registries, syndromic systems, wastewater monitoring, occupational surveillance, and emergency exemptions may be ethically authorized without individual informed consent when they are necessary for a legitimate public-health purpose, proportionate to that purpose, governed by safeguards against misuse, and oriented to the common good. These are long-standing conditions in public-health ethics, and they locate the authority for nonconsensual collection in public legitimacy rather than in the absence of objection [42,49-54]. Cancer registries, notifiable-disease reporting, and newborn screening rest on the same logic, and each carries a purpose limitation that the initial authorization was designed to serve.

The absence of consent does not mean the absence of authorization. What it means is that authorization rests on public legitimacy, statutory mandate, proportionality, transparency where feasible, safeguards against misuse, and the maintenance of trust. The continuity question is whether that structure still supports the present use. Data ethically collected for immunization tracking, outbreak detection, or communicable-disease control can lose authorization continuity when they are later used for a materially different purpose, because the justification that licensed collection without consent was tied to the original public-health aim and does not travel automatically to new aims [49,50]. The relevant test is not whether a legal basis can still be cited, but whether the public justification that licensed collection extends to the use now proposed.

Consider an immunization information system. The original system may be defensible without individual consent because immunization surveillance protects population health and supports continuity of care. Repurposing the same data for immigration enforcement changes the justificatory structure. The problem is not merely that consent was absent; it is that the new use no longer fits the expectations, proportionality, or trust relationship that made nonconsensual public-health collection legitimate, and that visible reversals of this kind can deter participation in the surveillance systems on which population health depends. Contextual integrity explains this type of failure. Privacy and authorization depend on context, actors, purposes, and transmission norms, and not only on the type of data at issue, a lesson reinforced by debates over the responsible use of digital data during public-health emergencies [13,42,49,55]. Investigative genetic genealogy, in which forensic actors query consumer or research genomic databases, is a further instance in which lawful technical access coexists with a serious break in authorization and relational continuity.

## Case Vignettes

### Overview

The following vignettes show the same inferential pattern across distinct data-stages, in each of which one continuity signal is visible and true while other domains are weak. They are chosen to span material, computational, organizational, and generative transformations, so that the pattern is not mistaken for a feature of any single technology. In each case the corrective action is the same in form even though the endangered domain differs: identify the signal doing the justificatory work, and test whether the other domains actually support the decision [8]. Their order moves from the most material transformation to the most abstract, so that the reader can watch the same inference recur as the object of governance becomes progressively less tangible.

### Polygenic Risk Scores

Polygenic risk scores show why the Continuity Trap must be distinguished from technical transportability. Linkage disequilibrium, allele-frequency differences, effect-size heterogeneity, and gene-environment interaction help explain why many scores perform differently across populations, and why scores developed largely in populations of European ancestry can transfer poorly to others. Those are technical and epidemiological problems. The Continuity Trap appears when a well-documented genome-wide association pipeline, ancestry adjustment, or validation workflow is treated as if it were sufficient governance warrant to move a score into new populations or clinical settings without reopening semantic and relational questions [56-58]. Treating a validated score as portable because its construction was rigorous confuses the reliability of the pipeline with the appropriateness of the categories and permissions it carries into a new setting.

Computational continuity is the persistence of technical reproducibility across analytic stages: the same pipelines, quality-control steps, ancestry adjustments, and validation procedures can be consistently applied. Governance continuity is different. It concerns whether categories, permissions, social assumptions, and relationships remain appropriate in new populations or deployment settings. Population descriptors such as African, European, Black, Yoruba, ethnicity, race, and ancestry do not do identical work across contexts, and treating them as stable technical variables can import social categories into clinical inference without warrant. Their responsible use requires explicit descriptor justification, limits on population claims, and documentation of context and uncertainty, so that a reproducible score is not mistaken for an interpretable or transferable one [59-62]. A descriptor that was a reasonable proxy in one population may encode a different construct in another, so semantic stability has to be argued rather than assumed.

### Legacy Biospecimens and Induced Pluripotent Stem Cell Derivation

Legacy biospecimens transformed into induced pluripotent stem cell lines reveal the trap in a limiting case. Here the material continuity is exceptionally strong: the derivative is biologically traceable to the donor and retains genomic continuity. That strength can mislead review bodies into treating the cell line as an ethically continuous extension of the original specimen. Yet reprogramming changes the horizon of possible use, extending it to organoids, gene-edited derivatives, disease models, screening platforms, repository distribution, and commercial applications that the original donors are unlikely to have contemplated [63-66]. The consent that governed a stored specimen was given for a bounded set of uses, and biological traceability does not extend that consent to uses the donor could not have anticipated.

After derivation and distribution, induced pluripotent stem cell lines may become indefinitely self-renewing and difficult to withdraw from global circulation, which weakens the practical meaning of consent-based withdrawal rights once lines have propagated across laboratories. The continuity issue is not that such research is impermissible. It is that strong biological continuity can conceal weak authorization and relational continuity. Sustained engagement, local scientific participation, benefit-oriented governance, clear derivative-use limits, and attention to community expectations can interrupt the trap, and dynamic-consent and dynamic-governance models have been proposed for exactly this reason [64-66]. Governance that engages the source community early is also more robust, because it does not depend on the assumption that a widely distributed line can later be recalled.

### Federated Learning

Federated learning privileges locality. The statement that raw data remain at the local institution is often true and privacy-relevant, and it has made multi-institutional collaboration feasible where data transfer would not be. It does not answer whether clinical records generated for care may legitimately function as distributed training inputs for another purpose, whether diagnostic categories are commensurable across sites, whether lower-resource institutions bear disproportionate burdens in producing a global model, or whether patients and contributing sites have recourse when downstream products are commercialized or deployed asymmetrically [67-70]. Locality answers a transfer question; it does not answer a purpose question, and the two are easy to conflate when the architecture is presented as privacy-preserving by design.

Differential privacy, secure aggregation, gradient auditing, and bring-code-to-data arrangements reduce confidentiality and transfer risks, and privacy-preserving federated methods have advanced quickly in medical imaging. Their limitation is domain specificity. They reduce risks in the provenance and confidentiality space; they cannot decide whether the new purpose is within clinical trust, whether labels mean the same thing at each site, or whether source institutions have relational standing. The assurance that the data never moved can therefore become a proxy for the quite different claim that the governance problem is solved [68,71]. Confidentiality and legitimacy are different goods, and a method can secure the first while leaving the second untouched.

### Health-Related Large Language Models

Health-related large language models intensify the Continuity Trap because the distance between input documentation and downstream influence is difficult to govern. A model trained on a governed, research-consented clinical dataset presents a different problem from one trained retrospectively on a large, opaque, cross-border body of data that happens to contain health-relevant information. The literature on stochastic parrots, World Health Organization guidance on large multimodal models, reviews of privacy and ethics in health large language models, and recent assessments of their clinical promise all stress that documentation, safety testing, and removability are necessary but incomplete governance instruments [43,72-75]. The governance difficulty is that influence in these systems is diffuse: no single training document determines an output, so tracing a downstream harm back to the conditions of its source is far harder than for a conventional dataset.

Developers may document broad categories of source material without showing how particular persons or communities influenced model behavior, how downstream harms can be traced to source conditions, or what route exists for contesting a use. Machine unlearning may improve technical removability, but technical removability is not governance adequacy, and the ability to delete a training record does not restore a person’s standing to object to how a deployed model behaves. The Continuity Trap lies in treating input documentation as if it were a sufficient account of authorization, relational standing, and accountability, which is why calls for regulatory oversight of these models emphasize postdeployment obligations rather than documentation alone [5,6,47,76,77]. Oversight therefore has to attach to the deployed system and its effects, not only to the provenance of its training corpus.

## Toward Trigger-Based Continuity Review

The response to the Continuity Trap should be rigorous but parsimonious. Continuity review should be triggered when the data-stage undergoes a qualitative transformation in purpose, scope, inferential reach, or derivative status (Textbox 2). Routine reuse within the scope contemplated by the original data-access agreement should not ordinarily trigger review. A replication study using the same variables and population descriptors, a new analysis within the same research program, or internal quality-improvement work that does not generate group-level claims or commercial outputs would usually fall outside the trigger set. The distinction is between quantitative extension and qualitative transformation, and it is meant to concentrate scarce review capacity where the relationship of entrustment is actually at risk [25,44]. Framing review around triggers also gives investigators advance notice of what will occasion scrutiny, which is fairer and more predictable than review that appears to depend on the disposition of an individual committee.

**Trigger conditions and continuity assessment questions.**

**Trigger conditions**

- Hard-to-reverse transformation of source data or material.
- Repurposing of descriptors across populations, sites, ontologies, or historical periods.
- Expanded uses, including commercialization, cross-border transfer, public-to-private transfer, or law-enforcement use.
- Outputs likely to generate group-level claims, sensitive inferences, deployment asymmetries, or reputational harms.
- Absence of a credible pathway for accountability, contestation, local validation, benefit-sharing, or meaningful withdrawal.

**Continuity assessment questions**

- What is the present data-stage?
- Which visible continuity signal is doing most of the justificatory work?
- What evidence supports provenance, semantic, authorization, and relational continuity?
- Which domain is weakest or most uncertain?
- What feasible safeguard is matched to that domain? What unresolved tension remains, and who will monitor it?

## The Continuity Statement

For triggered cases, investigators or developers should submit a short continuity statement together with existing ethics, data-access, material-transfer, repository, or AI governance documentation. The statement is not a self-certification. The secondary data users draft it; reviewers evaluate it and may disagree with the domain assessment. If a trigger condition is present and the investigator asserts that all domains are secure, reviewers should treat the mismatch itself as a reason for scrutiny, in the same spirit as documentation instruments that ask producers to state the intended and unintended uses of a dataset or model [5,6,11]. A continuity statement of a page or two, attached to what already exists, is enough to make the reviewer’s task tractable and the investigator’s reasoning legible.

The safeguard must match the endangered domain. If semantic continuity is weak, the response should be descriptor justification, local validation, uncertainty disclosure, subgroup performance evaluation, or limits on population claims. If authorization continuity is weak, the response may be renewed notice, reengagement, restriction on derivative applications, or explicit limitation on use. If relational standing is weak, appropriate responses include advisory review, joint oversight, benefit-sharing provisions, contestability pathways, local deployment conditions, or commercialization limits. If provenance itself is weak, lineage reconstruction and transformation auditing are the relevant safeguards. Adding a further generic form or a further layer of documentation, when the weak domain is semantic or relational, is precisely the failure the framework is designed to avoid [26,27,59-61]. The governing question at each step is not whether more can be documented, but whether the specific relationship that has weakened is the one the safeguard actually repairs. Textbox 3 presents a worked example of a continuity statement.

**A worked example of a continuity statement. Federated learning sepsis model.**

**Present data-stage**

- A federated model trained across 5 hospitals using local electronic health records to predict sepsis deterioration, with raw data remaining at each site and a global model shared across the network.

**Strongest domain**

- Provenance/locality. Each site can document local data sources, extraction scripts, access logs, aggregation governance, and secure aggregation procedures.

**Weakest domain**

- Semantic continuity. “Sepsis,” “suspected infection,” “organ dysfunction,” antibiotic timing, laboratory availability, and intensive care unit thresholds differ across hospitals.

**Authorization concern**

- Clinical data were generated for care. Network-level model training and possible licensing require explicit institutional justification and limits on secondary commercialization.

**Relational-standing concern**

- Smaller hospitals contribute data and bear implementation burdens but may have little influence over model updates, deployment thresholds, revenue sharing, or public communication of performance.

**Domain-matched safeguards**

- Require site-level label validation; report performance and calibration by site; restrict claims that the model is “network validated” until semantic heterogeneity is resolved; require network governance representation from all contributing sites; prohibit external commercialization without renewed authorization review.

**Review decision**

- Proceed with staged development, not full deployment. Approval is conditional on semantic validation and relational-governance safeguards, not additional provenance documentation alone.

## Institutional Roles and Burden

Continuity-sensitive governance should be distributed across the institutions that actually control access, deployment, and value capture. Investigators and developers should characterize the data-stage, identify the continuity signal on which they rely, justify their descriptors, map downstream uses, and propose domain-matched safeguards. Research ethics committees, data-access committees, repositories, material-transfer authorities, and AI governance bodies should evaluate whether the weakest domain has been correctly identified. Funders and journals can require disclosure of continuity triggers and safeguards. Contracts can carry derivative-use restrictions, downstream reporting obligations, joint-governance provisions, and benefit-sharing conditions [27,44-46,78]. Distributing the work in this way also keeps any single body from being held to account for a life course it never controlled, which is often what drives defensive overdocumentation.

The continuity statement should be concise and should reuse existing forms wherever possible, so that the framework lowers rather than raises the total burden of review by redirecting effort from generic paperwork to the domain under strain. No single committee can govern the entire life course of a dataset, model, or biospecimen. The framework therefore assigns tasks to the institution with practical control over the relevant data-stage: the repository for access, the review board for human-participant and authorization issues, the material-transfer office for derivative material, the AI governance body for deployment, and the contracting parties for downstream restrictions. Low-resource settings should not be asked to perform full sociological reconstruction for every request; triggered review should rely on consortium-level descriptor metadata, standing advisory structures, shared templates, and distributed review responsibilities, consistent with stewardship principles that make data findable, accessible, interoperable, and reusable without treating reuse as automatically authorized [25,78]. The aim is to relocate effort, not to add a stage, so that a triggered review spends its attention on the one or two domains that a routine filing would otherwise leave unexamined.

## Conclusion

The Continuity Trap names a recurrent failure in the ethical governance of data science health research: institutions infer too much from the continuity signal they can most easily verify. Provenance logs, repository approvals, consent documents, public-health authorities, model cards, and locality-preserving architectures all matter, and each answers a genuine governance question. None can certify ethical continuity alone. Ethical continuity is distributed across provenance, semantics, authorization, and relational standing, and these domains can diverge as data, materials, descriptors, models, and institutions change [10]. Recognizing that the domains are distinct is what keeps a single reassuring signal from being mistaken for the whole of ethical continuity.

The value of naming the trap is practical. It allows reviewers to distinguish routine secondary use from qualitative transformation; to identify which continuity domain is carrying too much justificatory weight; and to impose safeguards that match the domain under strain. That response is more precise than universal rereview and stronger than generic compliance documentation. Data science health research is often most vulnerable not when documentation is absent, but when documentation is so reassuring that institutions stop asking harder questions. Provenance should begin ethical review; it should not end it [12].

## Data Availability

No primary data were generated or analyzed in this study. All sources cited are publicly available.

## Abbreviations

HIPAA: US Health Insurance Portability and Accountability Act
NIST: US National Institute of Standards and Technology
